# Glomus tumor of the leg: a case report

**DOI:** 10.11604/pamj.2018.31.186.9706

**Published:** 2018-11-15

**Authors:** Mohamed Ali Sbai, Sofien Benzarti, Wafa Gharbi, Wadia Khoffi, Riadh Maalla

**Affiliations:** 1 Department of Orthopedic Surgery and Trauma, MT Maamouri Hospital, Nabeul, Tunisia; 2 Department of Pharmacology, MT Maamouri Hospital, Nabeul, Tunisia; 3 Department of Plastic Surgery, La Rabta Hospital, Tunis, Tunisia

**Keywords:** Glomus tumorlower limb, leg

## Abstract

Glomus tumors are uncommon benign tumors developing from the neuro-myo-arterial glomus body. They are typically located in the fingers. The extra-digital involvement is unusual and makes diagnosis difficult. Only few cases have been reported in literature. We report an exceptional case of a glomus tumor of the lower leg in a 65-year-old male. The diagnosis was clinically suspected and confirmed by a biopsy. Surgical excision gave immediate pain relief. The aim of this report is to make the surgical community more aware of this entity based on the analysis of our own experience and a review of the literature.

## Introduction

Glomus tumors (GTs) are rare, benign tumors arising from neuro-myo-arterial glomus bodies, accounting for less than 2% of soft tissue tumors [1], and a malignant transformation of this tumor with metastasis is possible but remains exceptional [2]. Typically, GT occurs in the hand and are relatively easily diagnosed following the classic triad of pain, tenderness, and hypersensitivity to cold. However, they may develop anywhere, and extra-digital GT, like the location in the leg, is much more difficult to diagnose. They are usually misdiagnosed and improperly treated, due to the absence of the typical symptoms. We report an exceptional case of extra-digital GT causing leg pain.

## Patient and observation

**Figure 1:**
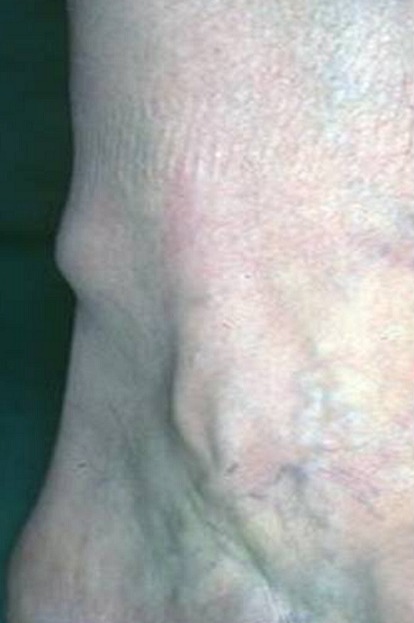
clinical appearance of the tumor of the leg

**Figure 2:**
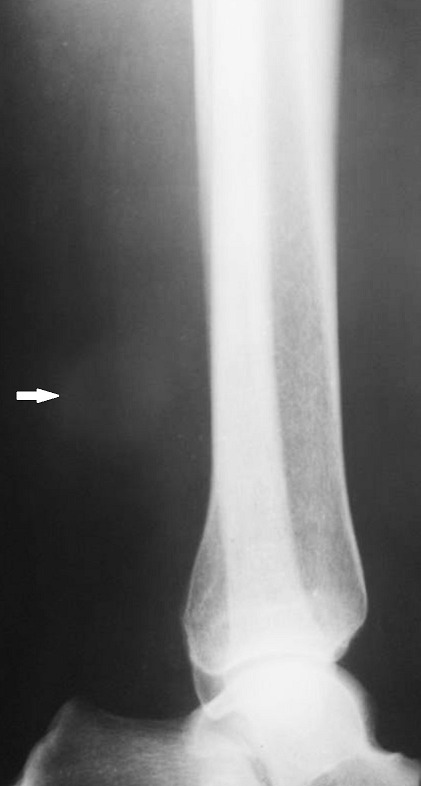
X-ray showing a swelling and densification of the soft tissues without bone injury (white arrow)

**Figure 3:**
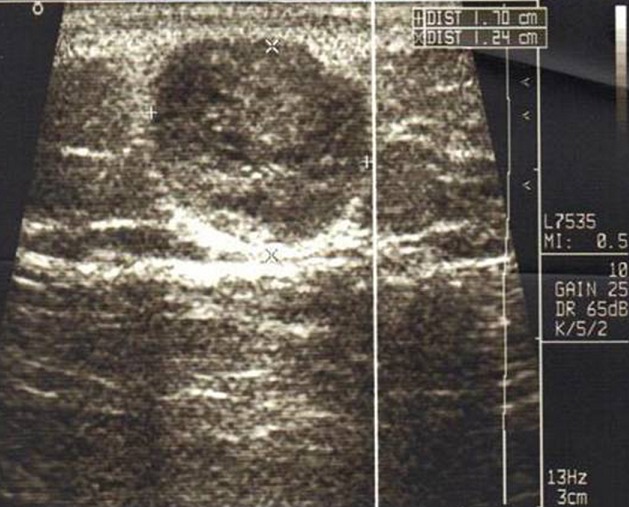
ultrasound image of the distal leg shows a hypoechoic, well-demarcated mass

**Figure 4:**
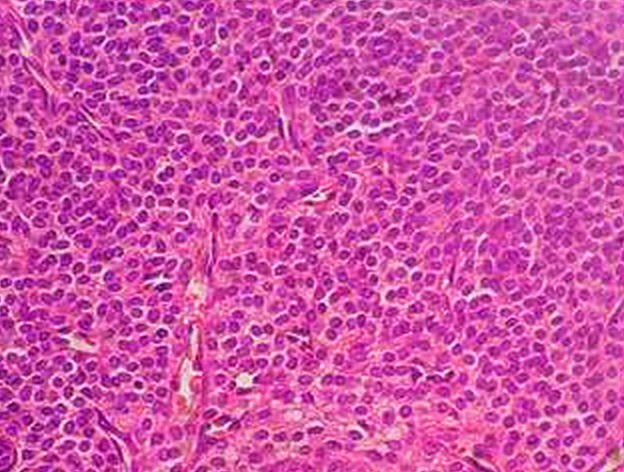
round and ovoid glomic cells (hematoxylin and eosin stain x 40)

A 65-year-old man presented with a painful swelling of his right lower leg. The pain started insidiously 6 months earlier, it was constant with hyperesthesia of the overlying skin, exacerbated by movements or upon palpation and reduced by rest and had increased in severity over the past 2 months. Physical examination revealed on palpation of the right lower leg a painful soft mass adherent to the deep layer measuring 2cm in diameter (Figure 1). The overlying skin was normal without any evidence of inflammation. Range of motion of the right leg was within normal limits. Neurological examination of the right lower limb was normal. Biology, including complete blood count, C-reactive protein and erythrocyte sedimentation rate, were within normal limits. Plain radiographs show thickening of the soft tissues without bone involvement (Figure 2). Ultrasound shows the presence of an echogenic, heterogeneous, rounded soft tissue mass, well demarcated measuring 17mm in diameter consisting with a nerve tumor (Figure 3). Surgical resection of the mass was performed and histological analysis concluded to a GT. A good outcome at 1 year post-operatively was observed with complete disappearance of pain without any evidence of recurrence (Figure 4).

## Discussion

GT are hamartomas developing from a neuro-myo-arterial structure localized at the dermo-hypodermic junction. The first clinical description of a GT was made in 1812 by Wood W [3]. GT are rare, mainly benign as blue-red painful tumors. These tumors account for 1% of all soft tissue tumors [3]. It is usually located in subungual region and lateral areas of the fingers. Extradigital GT are usually unexpected and difficult to diagnose. Unlike digital GT, which usually present with a triad of pain, tenderness to palpation, and intolerance to cold, extradigital GT have a more unusual clinical presentation and rarely present with hypersensitivity to cold [4].

The location in the leg is very rare [5-7], in a series of 56 patients with extradigital glomus tumors, the location was in the leg in only three cases [8], these benign tumors have a male-to-female predominance greater than 4 to 1 and can present at any time during adulthood [4]. Histologically, GT arise from the neuro-myo-arterial glomus body, which plays a role in the regulation of blood flow to the skin. There are three subtypes of GT, based on the pattern of differentiation and the degree of vascularisation. These include the solid type, which is the most frequent variant and is known as a ‘‘glomus tumor’’. These are characterized by sheets of eosinophilic, polygonal cells surrounding capillaries [8].

Plain radiography provides indirect information like a cortical erosion when the tumor is large, with thickening of the underlying soft tissues [9]. Ultrasonography is a useful tool showing a hypo-echoic well demarcaterd mass, hypervascularized on Doppler [10]. CT-scan, shows a soft tissue density mass that takes contrast. As for angiography, it confirms the vascular origin of the lesion, but remains invasive [11].

Magnetic Resonance Imaging (MRI), especially in high resolution represents the most sensible and specific tool distinguishing between the tumor itself and the normal tissue. It better defines its characteristics: indeed, it is a homogeneous well demarcated lesion, presenting as low signal intensity on T1-weighted images with rapid enhancement after gadolinium injection and high signal intensity on T2-weighted images. Finally, MRI provides accurate relations of the tumor with adjacent structures [9-11]. Treatment is surgical in solitary cases: complete resection including the joint capsule in articular locations to avoid recurrence. The outcome after surgical treatment is marked by a high recurrence rate: 1-18% depending on the series. However, spontaneous regression is possible. Malignant degeneration is rare [10].

## Conclusion

Glomus tumors are rare vascular tumors. The usual presentation is a solitary nodule in the distal portion of a finger, but can also occur everywhere, pain, hypersensitivity to cold and severe amyotrophy should raise suspicion for glomus tumors. Their diagnosis is histological. However, modern imaging tools, including high-resolution MRI allows a reliable diagnostic approach.

## Competing interests

The authors declare no conflict of interests.
